# Chronic Pharmacological and Safety Evaluation of Hematide™, a PEGylated Peptidic Erythropoiesis-Stimulating Agent, in Rodents

**DOI:** 10.1111/j.1742-7843.2008.00347.x

**Published:** 2009-02

**Authors:** Kathryn W Woodburn, Susan D Wilson, Kei-Lai Fong, Peter J Schatz, Charles B Spainhour, Daniel Norton

**Affiliations:** 1 Affymax Inc., Palo AltoCA USA; 2 Aclairo Pharmaceutical Development Group Inc., ViennaVA, USA; 3 Pennsylvania Biolab Inc., RadnorPA, USA; 4 Calvert Laboratories, OlyphantPA, USA

## Abstract

Hematide™ is a synthetic peptide-based, PEGylated erythropoiesis-stimulating agent, which is being developed for the chronic treatment of anaemia associated with chronic renal failure. To support the safety of long-term dosing of chronic renal failure patients, a comprehensive toxicology programme was implemented including rat subchronic and chronic studies. Rats were administered 0, 0.1, 1 and 10 mg/kg of Hematide every 3 weeks for 3 months via subcutaneous injection or for 6 months via intravenous injection. The dosing period was followed by a 6-week follow-up period. The primary pharmacology of Hematide resulted in erythroid polycythemia as measured by elevated haemoglobin levels that were time-and dose-dependent. The pharmacology profiles were similar regardless of administration route. For example, for male rats at Day 90, subcutaneous dosing resulted in haemoglobin increases of 2.7, 4.5 and 6.9 g/dl for 0.1, 1 and 10 mg Hematide/kg respectively, compared to 2.8, 5.7 and 7.4 g/dl increases for intravenous dosing. Histopathological changes were related to the prolonged severe polycythemia induced in normocythemic animals administered an erythropoiesis-stimulating agent. The findings included extramedullary haematopoiesis in the spleen and liver, bone marrow hypercellularity and organ congestion. Microscopic findings were reversible, demonstrating a return towards control findings within 6 weeks following cessation of dosing. Systemic exposures, based on both area under the curve (AUC) and maximum concentration (C_max_), were substantially greater for intravenous than subcutaneous administration. No Hematide-specific antibodies were detected. In conclusion, Hematide is a potent erythropoiesis-stimulating agent, and the studies provide support for the safety of clinical development, including chronic dosing, for the treatment of anaemia associated with chronic renal failure.

Anaemia in patients with chronic renal failure, which is caused primarily by an inadequate production of erythropoietin by the damaged kidneys [1], results in a reduction in length and quality of the patient's life [2]. The treatment of anaemia associated with kidney disease has been successfully carried out on more than a million patients by the use of recombinant human erythropoietin proteins [3]. However, the short half-life and pharmacological action of the currently available recombinant products, their potential to cause antibodies against endogenous erythropoietin and related analogues, high cost and lack of room temperature stability provide development incentives.

Hematide™ is a synthetic peptide-based, PEGylated erythropoiesis-stimulating agent. The molecule was designed to bind and activate the erythropoietin receptor, stimulating red blood cell production [4]. PEGylation generally results in an increase in protein/peptide solubility, an increase in plasma half-life by shielding the molecule from proteolysis and by reducing renal clearance, and a decrease in immunogenicity by masking epitopes [5]. Hematide's novel primary amino acid sequence is unrelated to that of human erythropoietin. Therefore, any potential antibody formed to Hematide is not likely to cross-react with erythropoietin and induce pure red cell aplasia. Hematide's lack of erythropoietin immunological cross-reactivity makes it capable of increasing haemoglobin in rats when their anaemia was caused by anti-erythropoietin antibodies that cross-react with endogenous erythropoietin following the administration of recombinant human erythropoietin [6]. Most importantly, it has eliminated the need for transfusions in patients with anti-erythropoietin antibody-mediated pure red cell aplasia [7].

Hematide is being developed for the treatment of anaemia secondary to chronic renal failure in a patient population that requires lifelong therapy. To support chronic clinical therapy, a series of repeat-dose pharmacodynamic, safety and pharmacokinetic studies was performed in animals, including normocythemic rats. Sprague–Dawley rats were studied after subcutaneous injections of Hematide every 3 weeks for 3 months and after intravenous administration every 3 weeks for up to 6 months.

## Materials and Methods

All animals received care in compliance with Guide for the Care and Use of Laboratory Animals (NIH Publication, 1996) and this study was conducted under the umbrella of an Internal Animal Care and Use Committee. Sprague–Dawley rats, approximately 8 weeks old and weighing 148–293 g at the start of dosing, were obtained from Harlan Sprague Dawley Inc. (Frederick, MD, USA). The 6-month intravenous study utilized doses of 0, 0.1, 1.0 and 10 mg/kg/dose administered every third week for up to 6 months for a total of 10 doses (Days 1, 22, 43, 64, 85, 106, 127, 148, 169 and 190). The low dose of 0.1 mg/kg was based, in part, from the results from the Phase I Hematide healthy volunteer study [8] in which participants received a single intravenous dose of Hematide (0.025, 0.05, or 0.1 mg/kg) or placebo. Hematide showed pharmacological activity characterized by a statistical increase in reticulocytes at all doses evaluated and a statistically and biologically significant increase in haemoglobin levels (approximately 1 g/dl) at 0.1 mg/kg that was sustained for longer than 1 month. Mid-dose of 1 and high dose of 10 mg/kg are 10 and 100 × multiples of the pharmacological active dose from the human healthy volunteer study. The study design incorporated an interim sacrifice on Day 90 (10/sex/group), a terminal sacrifice on Day 195/196 (20/sex/group) and a 6-week recovery period sacrifice on Day 232 (5/sex/group). For the 3-month subcutaneous study, Hematide was administered at doses of 0, 0.1, 1.0 or 10 mg/kg once every 3 weeks for a total of five doses (Days 1, 22, 43, 64 and 85). On Day 90 following the dosing period, the animals (10/sex/group) were killed. The remaining rats (5/sex/group) continued on study, untreated, following the final dose for a 6-week recovery period, at which time they were killed (Day 126/127). Hematide was formulated in 10 mM acetate buffered saline (pH 5.5) [4]. Doses were given as a single bolus 5 ml/kg injection.

A subset of rats for each study was utilized for pharmacokinetic analysis. Blood was collected from nine animals/sex/group (when applicable) at 9 time-points (3 per rat) starting on Days 1, 85 and 190 (intravenously only). Plasma drug concentration levels were determined and pharmacokinetic parameters were calculated.

Mortality and clinical observations were evaluated at least daily. Subcutaneous injection sites were scored according to Draize [9]. Body weights and food consumption were recorded weekly. Ophthalmology examinations were performed before treatment initiation and within 2 weeks prior to scheduled terminations. Upon termination, blood samples were collected for evaluation of haematology, coagulation and serum clinical chemistry parameters. Blood for additional evaluation of haematology was collected on Days 20, 41, 62, 104, 117/118 (subcutaneous), 125 (intravenous), 146 (intravenous), 167 (intravenous), 188 (intravenous) and 218 (intravenous). Urine was collected overnight for urinalyses on Day 1 and prior to each scheduled sacrifice from animals in Groups 1–4. Analysis of serum for the presence of anti-Hematide antibody was conducted on blood samples collected on Days 35, 83, 126/127 (subcutaneous), 195/196 (intravenous) and 232 (intravenous).

Determination of plasma drug levels and detection of anti-Hematide antibodies were performed by enzyme-linked immunosorbent assays (ELISA) as previously described [4,6,8]. Non-compartmental pharmacokinetic parameters were calculated from plasma concentration–time profiles of Hematide using WinNonlin® software (version 4.1, Pharsight, Mountain View, CA, USA).

Complete necropsies were performed, tissues were harvested, and organs were weighed. Due to mortality, primarily at the high dose, not all of the animals survived to scheduled sacrifice on Days 195/196 or 232. Tissues were harvested at each necropsy and for premature decedents, organs weighed and tissues evaluated microscopically.

### Statistical analyses

Data are expressed as mean with standard deviation. Comparisons of parameters were performed using a one-way analysis of variance followed by a post-hoc Dunnett's test. A P value of <0.05 was considered statistically significant.

## Results

### In-life evaluations – Hematide-associated effects

#### Clinical observations, dermal scoring, body weight, food consumption and ophthalmological evaluation

For the 6-month intravenous study, the most common clinical observation was dark pink to red discoloration of the paws and red staining on the cage paper and/or urogenital area. The findings were likely due to the sustained erythropoiesis induced by Hematide in normocythemic rats. The erythropoietic findings are discussed in greater detail in subsequent sections. For the 3-month subcutaneous study, there were generally no test article-related effects on clinical observations.

There were no test article-related effects on body weight or food consumption for the 0.1 and 1 mg/kg/dose groups in the 6-month intravenous study. Increased group body weight compared to concurrent controls was recorded on Day 36 and weekly from Days 57 to 169 for the 10 mg/kg/dose females. The increase in body weight correlated with an increase in food consumption. Conversely, 10 mg/kg/dose males had significantly decreased group mean body weight compared to concurrent controls on a weekly basis from Day 155 to Day 176. However, no significant changes in food consumption were observed. For the subcutaneous 3-month study, there were no test article-related effects on either body weight or food consumption.

For the intravenous study, there were no test article-related ophthalmological findings during the first 3 months, which is consistent with ophthalmological evaluations in rats dosed with Hematide by subcutaneous injection. A reversible trend observed during ophthalmic examination of the rats included an increase in vascular perfusion/hyperaemia of the choroidal and retinal vasculature across all groups, including controls, with increased severity in the mid-and high-dose animals.

#### Haematology, serum chemistry and urinalyses

**Fig. 1:**
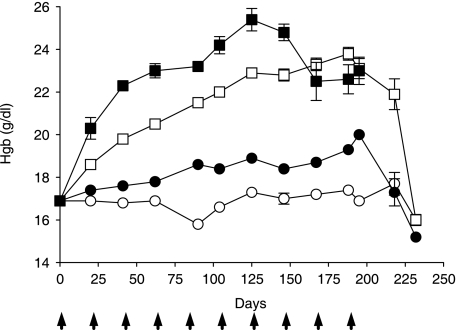
Dose-and time-dependent increases in haemoglobin (Hgb) after intravenous administration. Male rats received vehicle (○), 0.1 (∂), 1.0 (□) or 10 (▪) mg Hematide/kg/dose every 3 weeks for 6 months. Data are expressed as mean ± standard deviation with arrowheads denoting days of injection. For 0 through 1 mg/kg, 10 rats were sampled per dose per time-point through Day 188, Day 195 represented 18–20 animals while the remaining time-points represented 5 animals. Six to 10 rats were sampled per dose group for each time-point for the 10 mg/kg group.

Regardless of the route of administration (i.e. intravenous versus subcutaneous), Hematide administration resulted in erythropoiesis, which led to pronounced and sustained polycythemia at the 1 and 10 mg/kg doses. The effect of intravenous Hematide administration every 3 weeks for 6 months on haemoglobin levels in normocythemic male rats is depicted in fig. 1. In addition, the figure delineates the time-course for the reversibility of the Hematide-induced haemoglobin increases following the 6-week recovery period. By Day 125, the haemoglobin levels in the 0.1, 1, and 10 mg/kg groups were 18.9 ± 0.73, 22.9 ± 0.75, and 25.4 ± 1.40 g/dl, which reflect a increase of 1.6, 5.6, and 8.1 g Hgb/dl, respectively, over the concurrent vehicle control group (17.3 g/dl). The changes in haematology had reversed following cessation of treatment (Day 190).

**Fig. 2:**
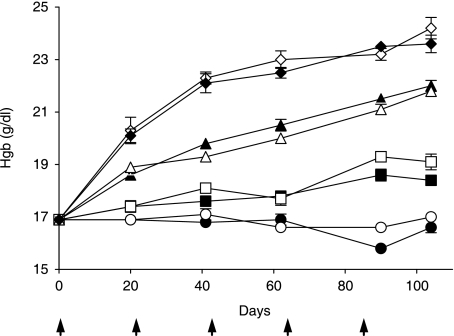
Comparable pharmacological response after intravenous and subcutaneous administration. Generation of mean haemoglobin (Hgb) ± standard deviation after intravenous (closed symbols) and (open symbols) subcutaneous administration every 3 weeks to male rats. Animals were dosed with vehicle (circles), 0.1 (squares), 1.0 (triangles), or 10 (diamonds) mg/kg. Arrows denote administration days. Each point represents the mean of 9–10 animals through Day 90 and 5–10 animals for Day 104.

Comparable pharmacological responses, with respect to haematopoiesis, were observed when Hematide was administered to rats via the subcutaneous or intravenous routes every 3 weeks for a total of five injections. Following administration of Hematide, there was a dose-dependent increase in haemoglobin level, the magnitude of which was similar between intravenous and subcutaneous administration at each respective dose level and for each respective time-point evaluated. Day 90 haemoglobin values in male rats are representative of the comparability in the pharmacological response for intravenous and subcutaneous administration of Hematide. Subcutaneous dosing resulted in haemoglobin increases of 2.7, 4.5 and 6.9 g/dl over concurrent controls for 0.1, 1 and 10 mg Hematide/kg, respectively, compared to 2.8, 5.7 and 7.4 g/dl increases, respectively, for intravenous dosing. Figure 2 illustrates the temporal relationship between haemoglobin increases and duration of dosing and number of doses following intravenous and subcutaneous Hematide administration.

**Fig. 3:**
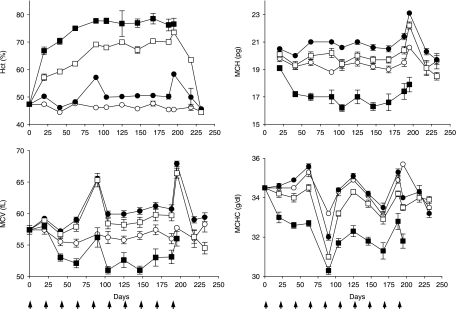
Hematide induces significant changes in haematocrit (Hct) and secondary haematologic indices. Alterations in Hct, mean corpuscular volume (MCV), mean corpuscular haemoglobin (MCH), mean corpuscular haemoglobin concentration (MCHC) were assessed after intravenous administration of vehicle (○), 0.1 (•), 1.0 (□) or 10 (▪) mg Hematide/kg every 3 weeks to female rats for 6 months. Mean data ± standard deviation is presented with arrows denoting days of administration. For 0 through 1 mg/kg, 9 to 10 rats were sampled per dose per time-point through Days 188, Day 195 represented 19–20 animals while the remaining time-points represented 4–5 animals. Five to 10 rats were sampled per dose group for each time-point for the 10 mg/kg group. The spikes on Days 85 and 195 are related to samples taken soon after dosing (5 days) compared to the other time-points which were generally taken 2 days preceding dosing.

The effect of Hematide on haemotocrit and red blood cells indices in female rats after intravenous administration every 3 weeks for 6 months is displayed in fig. 3. Changes were seen in all groups, but effects were most striking in the high-dose group (10 mg/kg). The changes are consistent with those observed in the male rats after intravenous dosing and in male and female rats after subcutaneous dosing. Hematide (intravenously and subcutaneously) also induced alterations in secondary haematological indices (mean corpuscular volume, mean corpuscular haemoglobin, mean corpuscular haemoglobin concentration) as well as red blood cell morphology (anisocytosis, polychromasia, macrocytosis) that were consistent with polycythemia. The high-dose groups produced smaller red blood cells (i.e. resulting in a decreased mean corpuscular volume), which contained less haemoglobin and haemoglobin concentration (i.e. resulting in a decreased mean corpuscular haemoglobin and mean corpuscular haemoglobin concentration, respectively). In contrast, the low (0.1 mg/kg) and mid-dose (1.0 mg/kg) groups produced larger red blood cells (mean corpuscular volume) with slightly more total haemoglobin per cell (mean corpuscular haemoglobin) but generally the same haemoglobin concentration (mean corpuscular haemoglobin concentration) as the concurrent vehicle control group. Haematological effects were similar to controls 6 weeks after cessation of dosing.

**Fig. 4:**
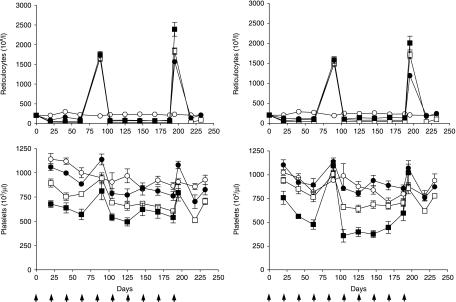
Reticulocyte and platelet profiles in female (left) and male rats (right) after intravenous administration of vehicle (○), 0.1 (•), 1.0 (□) or 10 (▪) mg Hematide/kg every 3 weeks for 10 administrations followed by a 6-week recovery. Data are represented as mean values ± standard deviation. Arrows denote days of administration. For 0 through 1 mg/kg, 8 to 10 rats were sampled per dose per time-point through Days 188, Day 195 represented 18–20 animals while the remaining time-points represented 4–5 animals. Five to 10 rats were sampled per dose group for each time-point for the 10 mg/kg group. Results show a synchronicity with dosing; when sampling times occurred soon after dosing (Days 90 and 195/196, 5 days after administration) elevations in parameters are observed.

Consistent with the stimulation of the red blood cell precursors and as expected for an erythropoiesis-stimulating agent, intravenous and subcutaneous Hematide induced reticulocytosis. The observed drug-induced changes on reticulocyte numbers were dependent upon sampling time in relationship to when the animals were dosed. Figure 4 depicts the changes in reticulocytes after intravenous administration. When blood samples were collected approximately 5–6 days after dose administration (terminal necropsies on Days 90 and 195/196), reticulocytes were significantly increased from controls at all Hematide dose levels. A compensatory decrease in reticulocytes, however, was observed when blood samples were collected approximately 3 weeks after dose administration. Reticulocyte numbers were typically less than control values 3 weeks after dosing, apparently in response to the increase in red blood cell numbers. Return of reticulocytes numbers towards vehicle-control values was observed following a 6-week recovery period in the groups administered 0.1 mg Hematide/kg, while rats at 1.0 mg Hematide/kg remained slightly decreased compared to concurrent controls in both sexes.

A synchronicity with dosing, similar to that noted for reticulocytes, was also observed with platelet levels in rats administered Hematide by intravenous (fig. 4) or subcutaneous injection. An increase in platelets was observed soon after dosing followed by a compensatory decrease approximately 3 weeks after dosing. The changes were related to Hematide administration; however, they were not of a magnitude considered to be toxicologically adverse and are conjectured to be related to an effect on megakaryocytes and/or precursors [10,11], or functional iron deficiency following accelerated reticulocytosis when high doses of erythropoiesis-stimulating agents are administered to normocythemic animals [12].

**Fig. 5:**
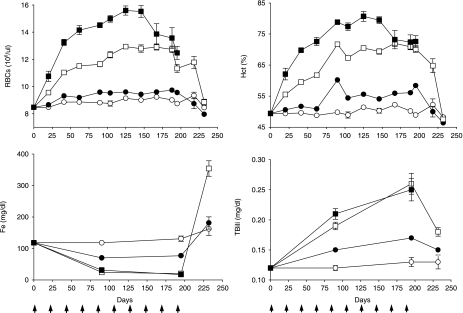
Iron and bilirubin profiles are consistent with accelerated erythropoiesis, polycythemia, increased red blood cell (RBC) turnover and subsequent recovery. Mean values ± standard deviation after intravenous administration of vehicle every 3 weeks (○), 0.1 (•), 1.0 (□) or 10 (▪) mg Hematide/kg to male rats for 6 months followed by a 6-week recovery. Arrows denote day of administration. Data for RBCs and haemotocrit (Hct) were obtained from the same animals represented in fig. 1. For Fe and bilirubin (TBili) determination, 0 through 1 mg/kg, 10 rats were sampled on Day 1, 9 to 10 on Day 90, 18 to 20 on Day 195 and 5 on Day 232. For the 10 mg/kg groups, 10 animals were sampled on Day 1, 1 on Day 90 and 7 on Day 195.

Alterations in serum chemistry were generally considered to be secondary to the exaggerated pharmacology of Hematide with findings being reversed after the 6-week recovery periods. Hematide-associated changes were generally similar after intravenous or subcutaneous dose administration. The alterations in serum chemistry included increases in bilirubin and aspartate aminotransferase and an initial decrease and subsequent increase in serum iron. Small increases, deemed toxicologically insignificant, in creatinine levels were observed at Day 195 in the mid-and high-dose groups (0.5 ± 0.09 mg/dl for 10 mg/kg males compared to 0.4 ± 0.02 mg/dl for concurrent controls; 0.6 ± 0.16 mg/dl for 10 mg/kg females compared to 0.5 ± 0.05 mg/dl for concurrent controls, P < 0.05). The changes in bilirubin and serum iron are depicted in fig. 5, and are considered to be secondary sequelae to the marked increases in red blood cells and haemotocrit. The increases in bilirubin and aspartate aminotransferase are likely due to increased erythrocyte turnover secondary to accelerated erythropoiesis and/or red blood cell haemolysis. The initial serum iron depletion on Days 90 and 195 is likely the result of incorporation into haemoglobin during rapid erythrocyte production (e.g. a functional iron deficiency). By Day 232, total serum iron was increased, which likely reflects the release of iron during erythrocyte breakdown secondary to significant polycythemia.

Urinalyses evaluation showed there were no differences in urine pH or specific gravity after approximately 3 months of treatment. On Day 90, there was an increase in urine protein compared to concurrent controls for male rats treated with ≥1.0 mg Hematide/kg after intravenous administration and for female rats receiving subcutaneous administration. On Day 126/127, three of five females dosed at 10 mg Hematide/kg subcutaneously had notably increased urine protein compared to controls. On Day 195/196, there were significantly decreased group urine pH values compared to concurrent controls for male rats intravenously dosed at ≥1.0 mg Hematide/kg. The changes in urine protein correlated with the renal glomerular and tubular lesions. There were no clear test article-related effects on qualitative or quantitative urinalysis parameters noted after a 6-week recovery.

Prothrombin time was significantly increased from controls in both the high-dose intravnous and subcutaneous males at Day 90. Activated partial thromboplastin time was increased in the high-dose animals at Day 90. By Day 195/196 of the intravenous toxicity study, both prothrombin time and activated partial thromboplastin time were significantly increased from controls for rats at dose levels equal to or greater than 1.0 mg Hematide/kg, except for the high-dose males. Although not attaining significance, the prothrombin time and activated partial thromboplastin time in the high-dose males were, however, notably increased. The observed increases in coagulation parameters were most likely spurious because similar false prolongations due to method interference have been reported in samples with high haematocrits [13].

#### Mortality

**Fig. 6:**
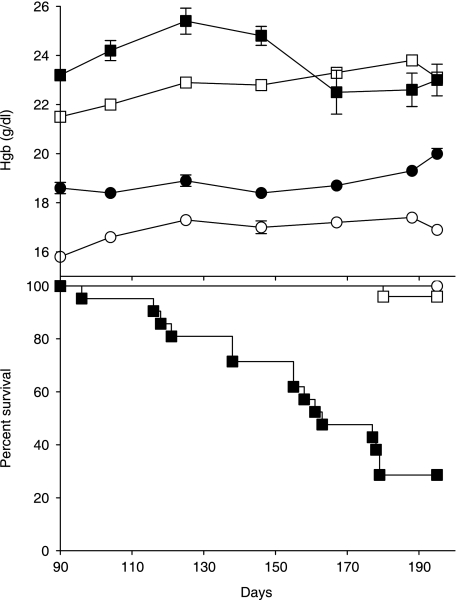
Relationship between polycythemia and mortality. Temporal generation of haemoglobin and survival after 6 months of Hematide dosing in male rats. The temporal generation of haemoglobin (Hgb) in male rats is shown in the top panel while survival (Days 90 to 190) is shown in the form of a Kaplan–Meier curve in the bottom panel. Hematide was administered intravenously at 0 (○), 0.1 (•), 1.0 (□) or 10 (▪) mg mg/kg every 3 weeks for a total ten administrations.

The physiological perturbations secondary to the sustained and profound polycythaemia that occurs when an erythropoiesis-stimulating agent is administered to normocythemic rats was considered to be the cause of death and unscheduled moribund sacrifices in the rats administered Hematide by either intravenous or subcutaneous injection. Premature deaths that were considered to be test article-related occurred in one mid-dose male rat (1.0 mg/kg; Day 50) from the 3-month subcutaneous study and 40 high-dose (10 mg/kg) and 2 mid-dose rats (1 mg/kg) from the 6-month intravenous study. Figure 6 illustrates the correlation between the incidence of mortality and the magnitude of haemoglobin increase and the dosing duration (i.e. duration of persistent polycythemia). The magnitude of the increase in haemoglobin associated with repeat dosing of Hematide exhibited a relatively sharp dose–response curve and appeared to plateau. The maximum increase in haemoglobin appeared to be approximately 7–9 g/dl. Mortality, which was greater in the intravenous versus subcutaneous study, was noted primarily after Day 90 of the study. The difference in mortality rate between the two studies, therefore, may in part reflect differences in the duration of the study (i.e. 90 versus 195/196 days for the subcutaneous versus intravenous study). In general, mortality was observed with the persistence of haemoglobin levels greater than approximately 23–24 g/dl for more than 90 days.

#### Immunogenicity

Antibodies to Hematide were not observed in any of the samples collected from the intravenous and subcutaneous studies.

#### Pharmacokinetics

Hematide exhibited persistence in the plasma following either intravenous or subcutaneous administration. With the exception of the 10 mg/kg subcutaneous dose on Day 1, the half-life of Hematide was generally similar after subcutaneous and intravenous administration at a given dose level. Half-life values ranged from 13.9 to 26.1 hr for doses of 0.1 and 1.0 mg/kg and, excluding the 10 mg/kg subcutaneous dose on Day 1, ranged from 29.7 to 40.6 hr at 10 mg/kg. Selected pharmacokinetic parameters are summarized in table 1.

**Table 1:** Pharmacokinetic parameters of Hematide following every 3 weeks subcutaneous and intravenous administration in rats for up to 6 months.

Day/sex	Doses	Route	Dose (mg/kg)	C_max_ (µg/ml)	AUC_0-inf_ (µg · hr/ml)	t_1/2_ (hr)
1/male	1	Intravenous	0.1	2.1	57.1	15.5
			1	21.6	810.8	19.9
			10	268.0	11324	31.3
1/male	1	Subcutaneous	0.1	0.274	12.8*	ND
			1	2.924	184.1	18.9
			10	50.87	3403	15.6
1/female	1	Intravenous	0.1	2.3	57.0	16.4
			1	22.7	684.7	20.1
			10	355.8	13769	40.6
1/female	1	Subcutaneous	0.1	0.279	13.6*	ND
			1	5.079	298.1	16.1
			10	54.32	4245	26.6
85/male	5	Intravenous	0.1	2.6	69.7	17.9
			1	30.1	1152	19.0
			10	314.3	14826	29.7
85/male	5	Subcutaneous	0.1	0.819	30.5	16.6
			1	3.807	245.1	26.1
			10	33.00	2460	37.1
85/female	5	Intravenous	0.1	1.8	62.4	18.7
			1	25.6	988.7	20.0
			10	310.7	15143	30.4
85/female	5	Subcutaneous	0.1	1.131	52.6	22.6
			1	8.875	589.7	22.0
			10	63.19	5622	33.0
190/male	10	Intravenous	0.1	1.9	56.1	13.9
			1	43.9	1314	17.0
190/female	10	Intravenous	0.1	2.7	62.3	17.2
			1	43.3	1285	17.0

* Insufficient data points to determine the terminal phase so AUC (72 hr) is expressed.

ND, not determined.

As anticipated, systemic exposures, based on both C_max_ and AUC, were substantially greater for intravenous versus subcutaneous routes of administration. After intravenous administration, C_max_was generally observed at the first sampling time (1 hr), but C_max_ after subcutaneous administration was within 24 to 48 hr after dosing (data not shown). Increases of C_max_ and exposure (AUC) values were greater than dose proportional across all dose levels and at all dosing intervals.

With intravenous administration, there were no remarkable gender-related differences in the pharmacokinetic parameters. On Day 85, gender differences were apparent after subcutaneous dosing, with C_max_ and AUC values twofold higher in females relative to males. Since the elimination half-life was not different between males and females, the gender-related exposure was suggestive of a difference in the extent of absorption after subcutaneous dosing. The subcutaneous bioavailability estimated at 1 mg/kg dose was 22.7% for males, and 43.4% for females.

In the 6-month intravenous study, there were no remarkable changes in pharmacokinetics at the low dose throughout the 6-month dosing period. A slight accumulation was apparent at the higher doses, which is considered likely to reflect the reduced plasma volumes secondary to haemoconcentration. No accumulation was observed at any dose after subcutaneous administration of Hematide every 3 weeks for 3 months.

### Post-mortem evaluations – Hematide-associated effects

#### Gross findings and organ weights

The most common test article-related gross findings were enlarged spleens and organ congestion. Organ congestion and/or increased erythropoiesis appeared to translate into an increase in mean organ weights for several organs including the liver and spleen. Injection site alterations were minimal and were not associated with any test article-related microscopic changes.

#### Histopathology

Histopathological changes observed in both the intravenous and subcutaneous studies were due to the primary pharmacological effects of Hematide and secondary sequelae. Primary pharmacological responses observed at scheduled necropsies (Days 90, 195/196) were indicative of increased red cell production and included hypercellularity of femoral bone marrow, increased erythropoiesis in the sternal bone marrow, extramedullary erythropoiesis in the spleen and liver, and congestion occurred in all Hematide treatment groups with dose-related severity. Such patterns were not observed following the 6-week recovery period.

Histopathological findings considered secondary to drug-induced haemodynamic changes included thrombosis and infarction in multiple organs primarily in the mid-and high-dose groups. In addition, cardiac changes, including thrombosis, stromal proliferation of the valves, myocardium or endocardium, and myocardial degeneration, were common in rats found dead and sacrificed moribund.

There were several findings in the rat that have been described as age-related spontaneous lesions, and appear to have been exacerbated by Hematide-related haemodynamic changes. The age-related spontaneous changes included chronic progressive nephropathy and/or tubular regeneration as well as periarteritis [14,15]. The periarteritis was considered likely related to altered haemodynamic patterns secondary to an increase in blood viscosity and haemoconcentration and was observed primarily in the high-dose group at the 3-month interim sacrifice and in the mid-and high-dose groups at 6 months. Haemodynamic alterations due to test article-induced polycythemia and haemoconcentration have been reported for other approved erythropoiesis-stimulating agents. The renal findings were attributed to altered renal perfusion secondary to Hematide-induced haemodynamic disturbances and/or the known renal toxic effects of excess haemoglobin (e.g. haemoglobin crystals and casts). Based on the severity of the renal changes, they are generally not anticipated to result in any significant functional perturbation.

## Discussion

Hematide is a potent erythropoiesis-stimulating agent, producing dose-and time-dependent increases in erythropoiesis. Unlike currently approved erythropoiesis-stimulating agent, no neutralizing antibodies were produced in the rodent studies, thereby allowing the first examination of the effects of chronic and severe polycythemia in normocythemic animals associated with administration of an erythropoiesis-stimulating agent.

The haematological and serum chemistry changes observed with Hematide, regardless of the route of administration (e.g. intravenous or subcutaneous) were anticipated based on the pharmacology of the drug. In addition, the test article-related effects noted, from discoloration of tissues, widespread congestion, organ weight changes, renal tubular morphological and functional alterations, thromboses with resultant ischaemic tissue damage and mortality, occurred in direct response to the pronounced erythropoiesis induced by Hematide's pharmacology in a normocythemic animal model. There were no tissue lesions that were considered to be related to a direct toxicity of Hematide. In surviving animals, the discontinuation of dosing led to recovery from the haematological and associated systemic effects and at least partial recovery for microscopic changes. The nature of the changes observed microscopically by the end of the recovery period is consistent with a full recovery over time.

As expected, the exposure based on C_max_ and AUC was substantively greater at comparable doses after intravenous versus subcutaneous administration. However, the findings (pharmacology and safety) in the subcutaneous rat study were similar to those from the intravenous studies. Furthermore, anti-Hematide antibody analysis after repeated dosing with Hematide by either the intravenous or subcutaneous route of administration did not demonstrate the presence of Hematide-reactive antibodies in rats that could have potentially confounded interpretation of the pharmacokinetic data.

## Conclusion

Hematide is presently being evaluated in clinical trials [16] for the treatment of anaemia associated with chronic renal failure. Hematide's clinical pharmacodynamic profile supports dosing every 3–4 weeks. The studies described herein were performed to support the use of Hematide in the clinical setting (i.e. chronic dosing). These studies were performed in healthy, normocythemic animals, with normal haemoglobin levels before treatment. The toxicological effects observed after intravenous and subcutaneous administration of Hematide are considered to be related to the exaggerated pharmacology and secondary sequelae that result from administration of an erythropoiesis-stimulating agent to a normocythemic animal. Chronic non-clinical studies can be conducted with Hematide without the confounding effects of neutralizing antibodies. The patient population for which chronic Hematide dosing is targeted will be anaemic and the intent of Hematide treatment is to return red blood cell parameters towards normal values. Therefore, the risk that the sequelae observed in the rat toxicity study, which are considered secondary to exaggerated pharmacology (e.g. sustained and pronounced polycythemia) will occur in the intended patient population is notably less.

## Conflict of Interest

K.W.W. and P.J.S. are employees of Affymax Inc., which is developing Hematide for the treatment of anaemia associated with chronic renal failure.
